# The long non-coding RNA lncRNA-DRNR enhances infectious bronchitis virus replication by targeting chicken JMJD6 and modulating interferon-stimulated genes expression via the JAK-STAT signalling pathway

**DOI:** 10.1186/s13567-024-01396-6

**Published:** 2024-11-05

**Authors:** Wenjun Yan, Xue Fu, Hao Li, Kailu Wang, Cailiang Song, Chengyao Hou, Cangwei Lei, Hongning Wang, Xin Yang

**Affiliations:** Key Laboratory of Bio-Resources and Eco-Environment, Ministry of Education, Animal Disease Prevention and Food Safety Key Laboratory of Sichuan Province, College of Life Science, Sichuan University, Chengdu, 610064 China

**Keywords:** Long non-coding RNA, infectious bronchitis virus, chJMJD6, interferon

## Abstract

Infectious bronchitis virus (IBV) is the causative agent of infectious bronchitis (IB), a severe disease that primarily affects young chickens and poses a significant challenge to the global poultry industry. Understanding the complex interaction between the virus and its host is vital for developing innovative antiviral strategies. Long non-coding RNA (lncRNA) plays a crucial role in regulating host antiviral immune responses. Our previous studies have shown that IBV infection disrupts the stability of lncRNA in host cells, indicating a potential regulatory role for lncRNA in IBV pathogenesis. It is still not clear how lncRNA precisely modulates IBV replication. In this study, we observed down-regulation ofMSTRG.26120.58 (named lncRNA-DRNR) expression in various chicken cell lines upon IBV infection. We demonstrated that silencing lncRNA-DRNR using siRNA enhances intracellular replication of IBV. Through exploring genes encoding proteins upstream and downstream of lncRNA-DRNR within a 100 kb range, we identified chJMJD6 (chicken JMJD6) as a potential target gene negatively regulated by lncRNA-DRNR expression levels. Furthermore, chJMJD6 inhibits STAT1 methylation, thereby affecting the induction of interferon-stimulated genes (ISGs) through the activation of the IFN-β-mediated JAK-STAT signalling pathway, ultimately promoting the intracellular replication of IBV. In summary, our findings reveal the critical role played by lncRNA-DRNR during IBV infection, providing novel insights into mechanisms underlying coronavirus-induced disruption in lncRNA stability.

## Introduction

Over the past two decades, coronaviruses (CoV) have caused three major pandemics affecting human societies. The most recent pandemic occurred in 2019 and had a profound impact on public health and the global economy [1]. Based on morphological and genomic characteristics, coronaviruses are classified into four genera: *Alphacoronavirus* (αCoV), *Betacoronavirus* (βCoV), *Gammacoronavirus* (γCoV), and *Deltacoronavirus* (δCoV) [2]. Notably, γCoV, with infectious bronchitis virus (IBV) as its representative pathogen, is recognised as the earliest identified coronavirus, predominantly infecting avian species such as chickens and turkeys [3]. IBV is an enveloped virus with a single-stranded, positive-sense, non-segmented RNA genome of approximately 27 kb in length [4]. The viral genome displays typical coronavirus features; two-thirds of the viral genomic sequence is dedicated to translating two polyproteins, PP1a and PP1ab, which are then cleaved by proteases into 15 non-structural proteins (nsp2 to nsp16). The genome's remaining third encodes four structural proteins: spike (S), membrane (M), envelope (E), and nucleocapsid (N), along with four accessory proteins (3a, 3b, 5a, and 5b), which contribute to the virus replication and pathogenesis [5]. When a pathogen infects the body, it triggers the host’s innate immune response [6]. IBV can intricately disrupt the host’s innate immune mechanisms at various levels, potentially impacting the induction of adaptive immune responses [7]. Specifically, IBV affects the type I interferon (IFN) pathway and its associated downstream signalling cascades [8, 9]. During the later stages of infection, IBV suppresses the expression of interferon-stimulated genes (ISGs) by inhibiting the phosphorylation and subsequent nuclear translocation of STAT1 [10]. Some ISGs encode proteins with natural antiviral properties, including ISG15, Mx1, viperin, and the RNA-dependent protein kinase induced by interferon. Moreover, numerous ISGs generate transcription factors that enhance the generation of interferons and other cytokines, thereby adjusting the immune response further [11, 12].

Non-coding RNAs (ncRNAs) consist of a diverse range of transcripts, including long non-coding RNAs (lncRNAs) and short ncRNAs such as microRNAs (miRNAs), PIWI-interacting RNAs (piRNAs), and small nuclear RNAs (snRNAs) [13]. lncRNAs are transcripts that exceed 200 nucleotides in length and cannot code for proteins. LncRNAs can interact with proteins, DNA, and other RNA molecules. This enables them to regulate chromatin structure and function, control the transcription of neighbouring (cis-acting) and distant (trans-acting) genes, modulate the assembly and activities of membraneless organelles, change the stability and translation of cytoplasmic mRNAs, and disrupt signalling pathways [14–16]. The primary way in which lncRNAs function is at the transcriptional level. LncRNAs can serve as molecular decoys, attracting transcription factors to promoters and enhancers, thus facilitating gene transcription and expression [17]. For example, the lncRNA NRON can interact with multiple proteins such as DYRK, CSE1L, CK1, and KPNB1. This interaction forms an RNA–protein complex that affects the activation and nuclear localisation of the NFAT1 transcription factor, ultimately suppressing transcription [18]. The lncRNA-DC binds to the transcription factor STAT3 in the cytoplasm of dendritic cells. This binding prevents the interaction between STAT3 and the protein phosphatase SHP1. As a result, the phosphorylation of the tyrosine-705 residue on STAT3 is protected, keeping STAT3 in an activated state [19].

There is growing evidence that the expression of host cell-encoded lncRNAs can change during viral infections. For example, infections by severe acute respiratory syndrome coronavirus 2 (SARS-CoV-2), human immunodeficiency virus (HIV), hepatitis C virus (HCV), influenza A virus (IAV), porcine epidemic diarrhea virus (PEDV), and porcine deltacoronavirus (PDCoV) have been found to affect the cellular lncRNA landscape [20–25]. These observations suggest that lncRNAs may play a role in reprogramming virus-host cell interactions. LncRNAs have been found to regulate viral infections through various mechanisms, including epigenetic regulation, modulation of viral latency, control of protein folding and subcellular localisation, and the alteration of alternative splicing patterns. For example, during IAV infection, the lncRNA-IVRPIE modulates the expression of cellular βinterferon and ISGs by influencing histone modifications, thereby contributing to the antiviral response [26]. Additionally, the lnc-zc3h7a can enhance the signal transduction of the RIG-I receptor and strengthen the TRIM25-mediated K63-linked ubiquitination of RIG-I, which is a critical innate immune sensor of viral RNA [27]. Furthermore, the lncRNA-ACOD1 can be transcriptionally induced by foot-and-mouth disease virus, herpes simplex virus 1, and vaccinia virus. It directly attaches to the substrate-binding site of glutamic-oxaloacetic transaminase 2 (GOT2) within viral inclusion bodies. This attachment enhances the catalytic activity of GOT2, thus promoting viral replication [28]. These examples illustrate the diverse regulatory roles of lncRNAs in modulating various stages of the viral life cycle and host antiviral defences, highlighting their importance as critical mediators of virus-host interactions.

In our previous study, we found that IBV infection can cause changes in the lncRNA expression profile of the chicken macrophage cell line HD11 [29]. We focused on one particular lncRNA, called lncRNA-DRNR, which was significantly altered. We discovered that it can inhibit viral replication by reducing the expression of a protein called chJMJD6. This study found that chJMJD6 can hinder the production of IFN-β and its downstream ISGs by dephosphorylating the transcription factor STAT1. This action suppresses the host’s antiviral response and helps the virus replicate. Our findings indicate that the lncRNA-DRNR may function as an upstream regulator of chJMJD6, suggesting that chJMJD6 may play a crucial role in the interaction between the host and the IBV virus during infection. These insights may offer new perspectives for developing targeted treatment strategies to manage IBV infections in poultry.

## Materials and methods

### Cell lines and virus

The HD11, DF-1, and LMH cell lines were cultured in a humidified atmosphere containing 5% CO2 at 37 °C. The cells were maintained in Dulbecco’s Modified Eagle Medium (DMEM) supplemented with 10% (v/v) foetal bovine serum (FBS, Gibco) and 1% penicillin–streptomycin (Gibco). The IBV strain Beaudette was stored in our laboratory, with a viral titre of 10^5.5^ TCID_50_/100 μL in DF-1 cells.

### RNA isolation, reverse transcription and qPCR

Total RNA was extracted from the cells using the RNAiso Plus RNA isolation kit (Takara, Dalian, China), following the manufacturer's instructions. 1 μg of the isolated total RNA was subjected to reverse transcription using the Evo M-MLV reverse transcription premix kit (Accurate Biology, Hunan, China) kit, as per the protocol provided by the manufacturer. Quantitative real-time PCR (qPCR) was then performed with the primer sequences listed in Table 1. The relative expression levels of the target genes were calculated using the 2^−ΔΔCt^ method, with GAPDH serving as the internal control. At various points following IBV infection, supernatants were harvested and a sequential tenfold dilution series from 10^–1^ to 10^–20^ was conducted. DF-1 cells were seeded in a 96-well microtiter plate. Then, the cells were infected with the prepared virus dilutions (100 µL per well), ensuring that eight replicate wells represented each dilution. Eight wells without virus infection were included within each plate as negative controls. The cell plates were incubated in the cell culture incubator and monitored daily for cytopathic effects (CPE) onset across all dilutions. After an incubation period of 5–7 days, the 50% Tissue Culture Infectious Dose (TCID_50_) was calculated using the Reed-Muench method.

**Table 1:** **Primers used for plasmid construction**

Primer name or use	Direction	Sequence
MSTRG.26120.58 or lncRNA-DRNR (qPCR)	F	ACGACATTAGGCGGTACGGAAT
	R	GAGGCTGGAGTGGCACAAGA
MSTRG.25256.5 (qPCR)	F	AGAAGTGTCTGGAGATGTC
	R	TGTTCCACACTGATGTCA
MSTRG.2137.5 (qPCR)	F	ACCGCCTAAAGAGTTACC
	R	CTTTGTTTATTTCTCTGTCCCT
MSTRG.6458.14 (qPCR)	F	GGTGTGGCTGGTGGACTGTA
	R	AGCCGCACCTGTAGTGAGAC
MSTRG.25416.43 (qPCR)	F	ATAAGAGGAGCAGAACAGTTA
	R	CTTCTTCTCCAGTAAACAACATA
IBV-N (qPCR)	F	TGCTGCTAAGGGTGCTGATACT
	R	AGGTCCGCCATCCGAGAATC
U1 (qPCR)	F	AGCACCACATAGCAATAATG
	R	CCTTCTAATTCCCTCAGTGA
GAPDH (qPCR)	F	ACTGTCAAGGCTGAGAACGG
	R	GCTGAGGGAGCTGAGATGA
Lnc-siRNA-1	F	ACGACATTAGGCGGTACGGAAT
	R	AUUUCCAAUAGCAUGCGUGTT
Lnc-siRNA-2	F	CAGGAGCAUAGCCAUAUCUTT
	R	AGAUAUGGCUAUGCUCCUGTT
Lnc-siRNA-3	F	GGCAGAUGGAUAUUCAGGUTT
	R	ACCUGAAUAUCCAUCUGCCTT
Lnc-siRNA-NC	F	GCGACGAUCUGCCUAAGAU
	R	AUCUUAGGCAGAUCGUCGC
chJMJD6-si-1	F	GCCCGCUGUACAUAUUUGATT
	R	UCAAAUAUGUACAGCGGGCTT
chJMJD6-si-2	F	CGUGGUUCAACGUCAUAUATT
	R	UAUAUGACGUUGAACCACGTT
chJMJD6-si-3	F	GGGUAACGGUGAUACAACUTT
	R	AGUUGUAUCACCGUUACCCTT
IFN-β (qPCR)	F	GCTCTCACCACCACCTTCTC
	R	GCTTGCTTCTTGTCCTTGCT
chIFITM3 (qPCR)	F	ATCGCAAAGTCCTGGGTG
	R	TGCTGCTGGTGGTTGAAGA
chMx1 (qPCR)	F	GGTGTCATTACTCGCTGT
	R	CTTTCTTCACCTCTGATGC

### Fluorescence in situ hybridisation (FISH)

Based on the known sequence of lncRNA-DRNR obtained from RNA sequencing, a specific FISH probe was designed and labelled with a Cy3 fluorophore. The HD11 cells were evenly seeded in a 6-well plate and cultured overnight. The cells were washed with pre-chilled PBS for 5 min, fixed with 4% paraformaldehyde at room temperature for 20 min, and permeabilized with pre-chilled permeabilization solution at 4 °C for 5 min, followed by PBS washes. The cells were hybridised with the Cy3-labeled probe targeting lncRNA-DRNR overnight at 42 °C and washed with PBS thrice. After blocking with rabbit serum at room temperature for 30 min, the cells were incubated with anti-DIG-HRP at 37 °C for 50 min, followed by PBS washes (three times, 5 min each). The Cy3 signal was then amplified using the Cy3-TSA reagent in the dark at room temperature for 5 min, followed by another set of PBS washes. Finally, the cell nuclei were counterstained with DAPI for 10 min in the dark, and the samples were observed under an inverted fluorescence microscope Olympus SpinFV-COMB.

### Cell component separation

HD11 cells (7 × 10^6^) were harvested and lysed for 15 min at 0 °C in 0.5% NP-40 lysis buffer containing the RNase inhibitor SUPERase (MCE, China). The cell lysates were centrifuged at 8000 rpm for 1 min at 4 °C. The supernatant, representing the cytoplasmic fraction, was transferred to a new microcentrifuge tube. The nuclear pellet was washed with 0.05% NP-40 buffer and centrifuged at 8000 rpm for 1 min at 4 °C to obtain the nuclear extracts. Finally, the cytoplasmic and nuclear extracts were purified and analysed by RT-qPCR. The relative abundance of the cytoplasmic and nuclear fractions was calculated by normalising the expression levels to the total (cytoplasmic + nuclear) expression, which was set as 1.

### Construction of chJMJD6 overexpressing plasmid

Based on the chJMJD6 sequence (NCBI Accession No.: NC_052549.1), an HA-tag was added to the C-terminus and the construct was cloned into the pcDNA3.1( +) expression vector. 100 μL of the bacterial culture harbouring the pcDNA3.1-chJMJD6-HA plasmid or the empty pcDNA3.1 vector was inoculated into 20 mL of LB medium supplemented with 10 μg/mL ampicillin. The cultures were then incubated at 37 °C with shaking at 250 rpm for 18–24 h. Plasmid DNA was extracted from the bacterial cultures using the SteadyPure non-endotoxin plasmid extraction kit (Accurate Biology, China), following the manufacturer’s instructions.

### siRNA design and transfection

Using the RNAi Explorer™ tool, we designed three siRNA sequences targeting lncRNA-DRNR and three targeting the chJMJD6 mRNA (sequences shown in Table 1). The siRNAs were synthesised and purified by high-performance liquid chromatography (HPLC) at Synbio Technologies, China. The synthesised siRNAs or the pcDNA3.1-chJMJD6-HA expression vector were transfected into HD11 cells at 70% confluency using Lipofectamine 3000 transfection reagent. Non-targeting siRNA (siCtrl) and the empty pcDNA3.1 vector were used as negative controls for normalisation and comparison with the specific knockdown and overexpression conditions. For the infection experiments, at 24 h post-transfection, the cells were infected with the IBV strain at a multiplicity of infection (MOI) of 1. Cells with or without IBV infection were harvested at designated time points for subsequent RNA or protein analysis.

### Western blot analysis (WB)

Proteins were extracted from cells by incubation with a detergent-containing lysis buffer (20 mM Tris–HCl, 150 mM NaCl, 1% NP-40, 1 mM EDTA, 5 mM MgCl2, 10% glycerol, and 1 mMPMSF). The cells were lysed on ice for 10 min, followed by centrifugation at 12000 rpm for 5–10 min. The supernatant was collected and mixed with protein loading buffer, then boiled at 95 °C for 10 min. The protein samples were separated by SDS-PAGE using 10% Bis–Tris gels and then transferred onto PVDF membranes. The membranes were blocked in PBST solution containing 5% (w/v) non-fat dry milk and 0.05% Tween 20 and shaken at room temperature for 1 h. The membranes were then incubated overnight at 4 °C with primary antibodies (anti-GAPDH, Proteintech, cat No.:60004-1-Ig; anti-IBV N protein, NOVUS, cat No.: NBP2-31102; anti-HA tag, Proteintech, cat No.:81290-1-RR, and anti-JMJD6, Proteintech, cat No.:16476-1-AP) diluted 1:500 to 1:5000 according to the manufacturer's recommendations. After three washes with PBST, the membranes were incubated with goat anti-mouse or anti-rabbit secondary antibodies for 45 min at 37 °C. The membranes were rewashed and treated with SuperKine™ Universal ECL chemiluminescence Solution (Abbkine, cat No.:BMP3010). The bound proteins were visualised using a BioRad Protein colour development system.

### Retrieval and multiple sequence alignment of JMJD6 amino acid sequences of various species

The amino acid sequences of JMJD6 from the three mammalian species (Human, Mouse, and Chicken) in FASTA format were retrieved from The National Center for Biotechnology Information (NCBI) database, with the accession numbers shown in Figure 4B. The online tool Clustal Omega was used with its default parameters for multiple alignments of the three JMJD6 sequences.

### Immunoprecipitation

Cells were transfected with the chJMJD6 overexpression vector or chJMJD6-specific siRNA for 24 h, then infected with IBV at a MOI = 2. After 24 h of viral infection, samples were collected according to the WB protocol. The cell culture supernatants were incubated overnight at 4 °C with the appropriate primary antibodies or rabbit IgG as a negative control under gentle agitation to allow antibody-antigen complexes to form. Then, 20 μL of magnetic beads were added per 500 μL of sample, and the magnetic beads were captured and washed three times with 1 × TBS. The antibody-antigen complexes were then incubated with the magnetic beads for 2 h at room temperature. After the incubation, the magnetic beads were separated using a magnetic rack for 10 s, the supernatant was discarded, and the beads were resuspended in lysis buffer containing protease inhibitors. This washing step was repeated three times. Finally, the bound proteins were eluted by adding SDS-PAGE loading buffer at a ratio of 100 μL per 20 μL of magnetic beads, heated at 95 °C for 10 min, and the supernatant was collected for WB analysis after magnetic separation.

### Indirect immunofluorescence staining

When the cell confluence reached approximately 70%, the HD11 cells were transfected with either the pcDNA3.1-chJMJD6-HA expression vector or the empty pcDNA3.1 vector. After 24 h, the culture medium was removed, and the cells were washed thrice with PBS. The cells were then fixed with 4% paraformaldehyde at room temperature for 15 min. Following fixation, the cells were washed three times with PBS and blocked with PBS containing 3% bovine serum albumin (BSA) for 1 h at 37 °C. The cells were then incubated overnight at 4 °C with the primary antibody. After three washes with PBS, the cells were incubated with the corresponding fluorescently-labelled secondary antibody for 1 h at room temperature. Finally, the cell nuclei were stained with DAPI for 10 min at room temperature, followed by three additional PBS washes. The stained cells were observed under an inverted fluorescence microscope.

### Statistical analysis

All data were analysed using GraphPad Prism 5.0. Differences were evaluated for statistical significance using one-way analysis of variance (ANOVA) or Student’s *t* test. *p* < 0.05 (*) indicates statistically significant, *p* < 0.01 (**) suggests statistically very significant, while *p* < 0.001 (***) implies statistically extremely significant.

## Results

### lncRNA-DRNR is down-regulated in IBV-infected DF-1, HD11, and LMH cells

Numerous studies have shown that viral infections can cause the dysregulation of host cell-encoded lncRNAs. Subsequently, viruses can exploit these differentially expressed lncRNAs to suppress the host’s innate antiviral immune responses, thereby promoting their survival and replication [20, 30]. Our prior investigation discovered that IBV infection disrupted lncRNA homeostasis in the chicken macrophage cell line HD11. Specifically, we found 181 lncRNAs with significant expression changes: 59 up-regulated and 122 down-regulated. Notably, the down-regulated lncRNAs comprised up to 67.4% (122/181) of the dysregulated lncRNA population [29]. This observation indicated that most of the modified lncRNAs may act as inhibitors of viral replication. The significant down-regulation in host lncRNAs following IBV infection suggests that these lncRNAs probably have crucial functions in limiting viral spread. In contrast, the virus may use the up-regulated IncRNAs to bypass the host's antiviral defences and support its own proliferation.

**Figure 1:**
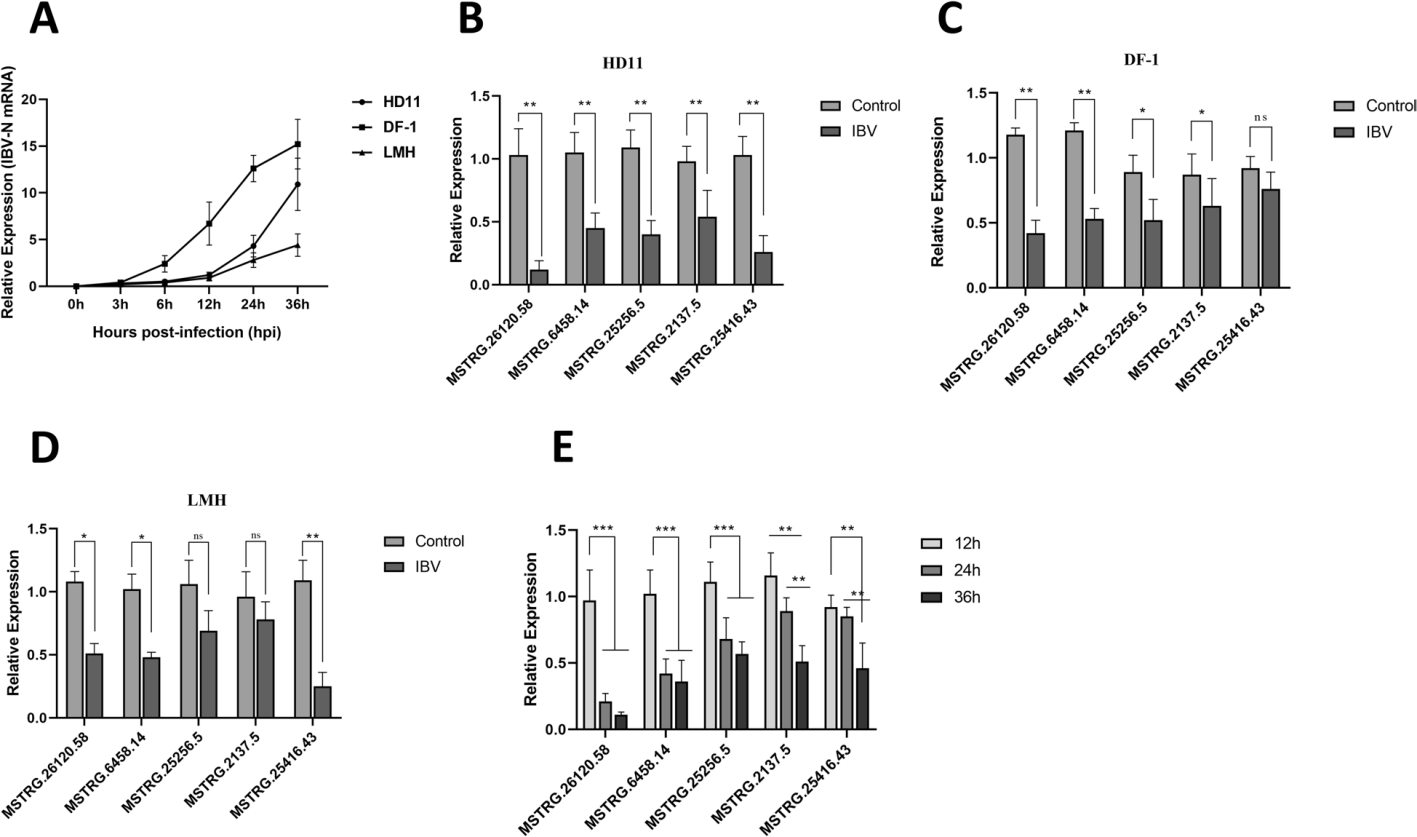
**Quantitative PCR (qPCR) validation of lncRNA down-regulation induced by viral infection.** **A** Analysis of the proliferation of the IBV-N gene in HD11, DF-1, and LMH cells. **B** Expression changes of the candidate lncRNA in HD11 cells following IBV infection at 24 hpi. **C** Expression changes of the candidate lncRNA in DF-1 cells following IBV infection at 24 hpi. **D** Expression changes of the candidate lncRNA in LMH cells following IBV infection at 24 hpi. **E** Changes in the expression levels of the candidate lncRNA at different time points post-IBV infection.

To investigate the roles of these significantly down-regulated lncRNAs in viral replication and identify potential antiviral target candidates, we used RT-qPCR to examine the expression changes of five selected lncRNAs before and after virus infection. The RT-qPCR analysis confirmed an increase in the viral gene expression level (IBV-N) during the infection in three cell lines: HD11, DF-1, and LMH (Figure 1A). After 24 h post-infection (hpi), we observed a significant down-regulation of all five candidate lncRNAs in HD11 cells following IBV infection, consistent with the previous RNA-seq results (Figure 1B). Afterwards, we confirmed the changes in expression of the potential lncRNAs following IBV infection in two more cell lines, DF-1 and LMH cells (Figures 1C and D). The findings indicated that in IBV-infected DF-1 cells, all IncRNAs except for MSTRG.25416.43 showed significant down-regulation. In IBV-infected LMH cells, MSTRG.25256.5 and MSTRG.2137.5 did not exhibit significant down-regulation, whereas the other three lncRNAs showed significant down-regulation. Furthermore, we infected HD11 cells with IBV at an MOI of 2 and collected cell samples at different time points post-infection (12 hpi, 24 hpi, and 36 hpi). Among the five candidate lncRNAs, the expression levels of MSTRG.26120.58 (named lncRNA-DRNR) and LNC_MSTRG.6458.14 were significantly down-regulated at both 24 hpi and 36 hpi compared to the 12 hpi (*p* < 0.01), while MSTRG.25256.5, MSTRG.2137.5, and MSTRG.25416.43 exhibited significant down-regulation only at 36 hpi (Figure 1E). In summary, we discovered that the expression of lncRNA-DRNR was significantly down-regulated in correlation with the IBV replication cycle. This down-regulation was consistent across different cell lines and was induced by IBV infection, indicating its potential role in regulating viral replication. As a result, we chose lncRNA-DRNR for further functional investigations.

### lncRNA-DRNR is evenly distributed in the cytoplasm and nucleus

**Figure 2:**
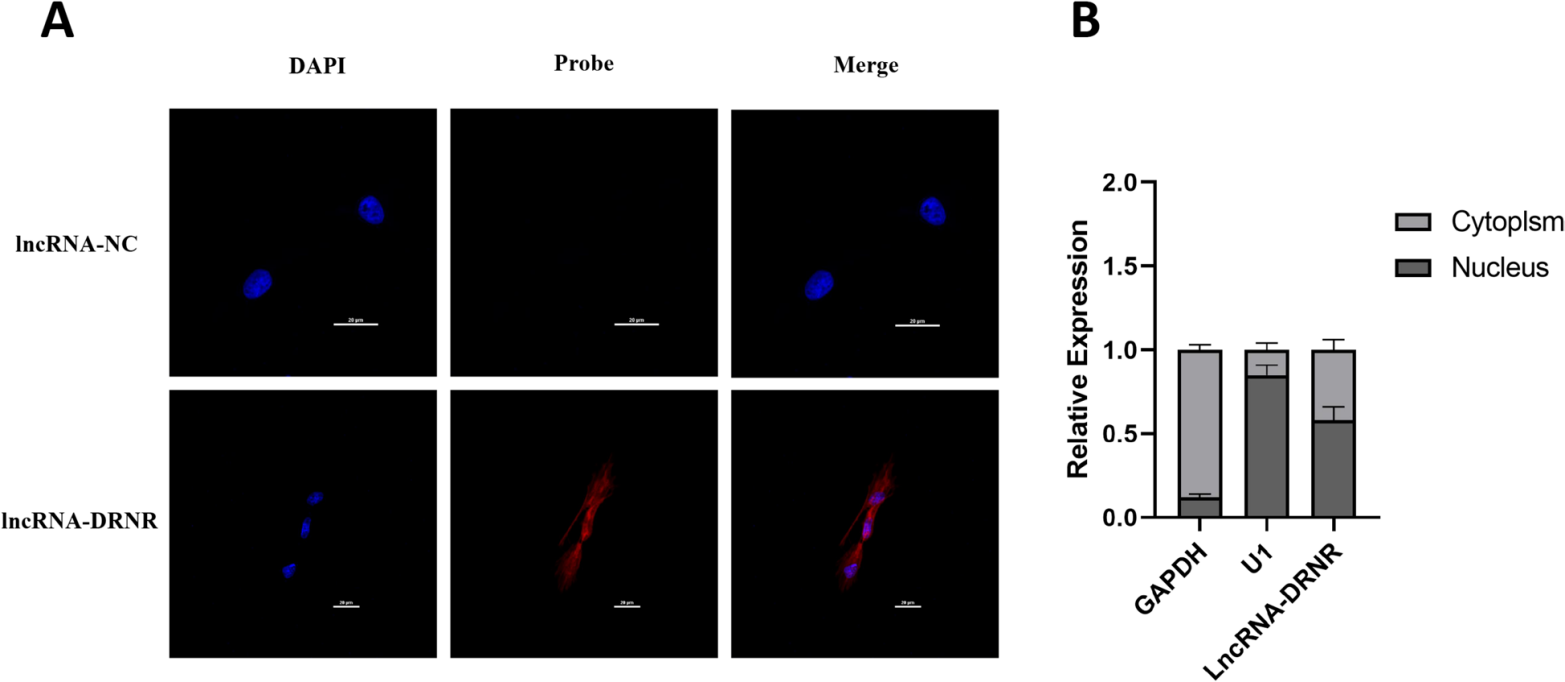
**Subcellular localisation of lncRNA-DRNR.** **A** FISH analysis to determine the subcellular distribution of lncRNA-DRNR. **B** Nuclear-cytoplasmic fractionation experiment to further validate the subcellular localisation of lncRNA-DRNR.

The subcellular localisation patterns of lncRNAs are closely associated with their diverse cellular activities and biological functions. Distinct subcellular localisations of lncRNAs may confer different cellular roles [31]. To gain a deeper understanding of the subcellular distribution of lncRNA-DRNR, we designed specific antisense oligonucleotide probes targeting lncRNA-DRNR, as well as corresponding digoxin-labelled secondary probes to amplify the hybridisation signals. We performed FISH and cellular fractionation experiments using IBV-infected HD11 cells to determine the subcellular localisation of lncRNA-DRNR. In addition, we used GAPDH (mainly present in the cytoplasm) and U1 (primarily present in the nucleus) as controls. The FISH results showed that the red fluorescence signals representing lncRNA-DRNR were detected in both the cell nucleus and the cytoplasm (Figure 2A). Furthermore, the cellular fractionation analysis revealed that lncRNA-DRNR was distributed with 58.4% in the nuclear fraction and 41.6% in the cytoplasmic fraction (Figure 2B). This indicates a relatively even distribution between the nucleus and cytoplasm. These findings suggest that lncRNA-DRNR may perform cellular functions through nuclear and cytoplasmic mechanisms. The equal distribution of lncRNA-DRNR within the cell indicates that it may play a role in regulating various cellular processes. This warrants further investigation into its specific functions, particularly in the context of viral infection.

### siRNA interferes with lncRNA-DRNR to promote IBV proliferation in HD11 cells

**Figure 3:**
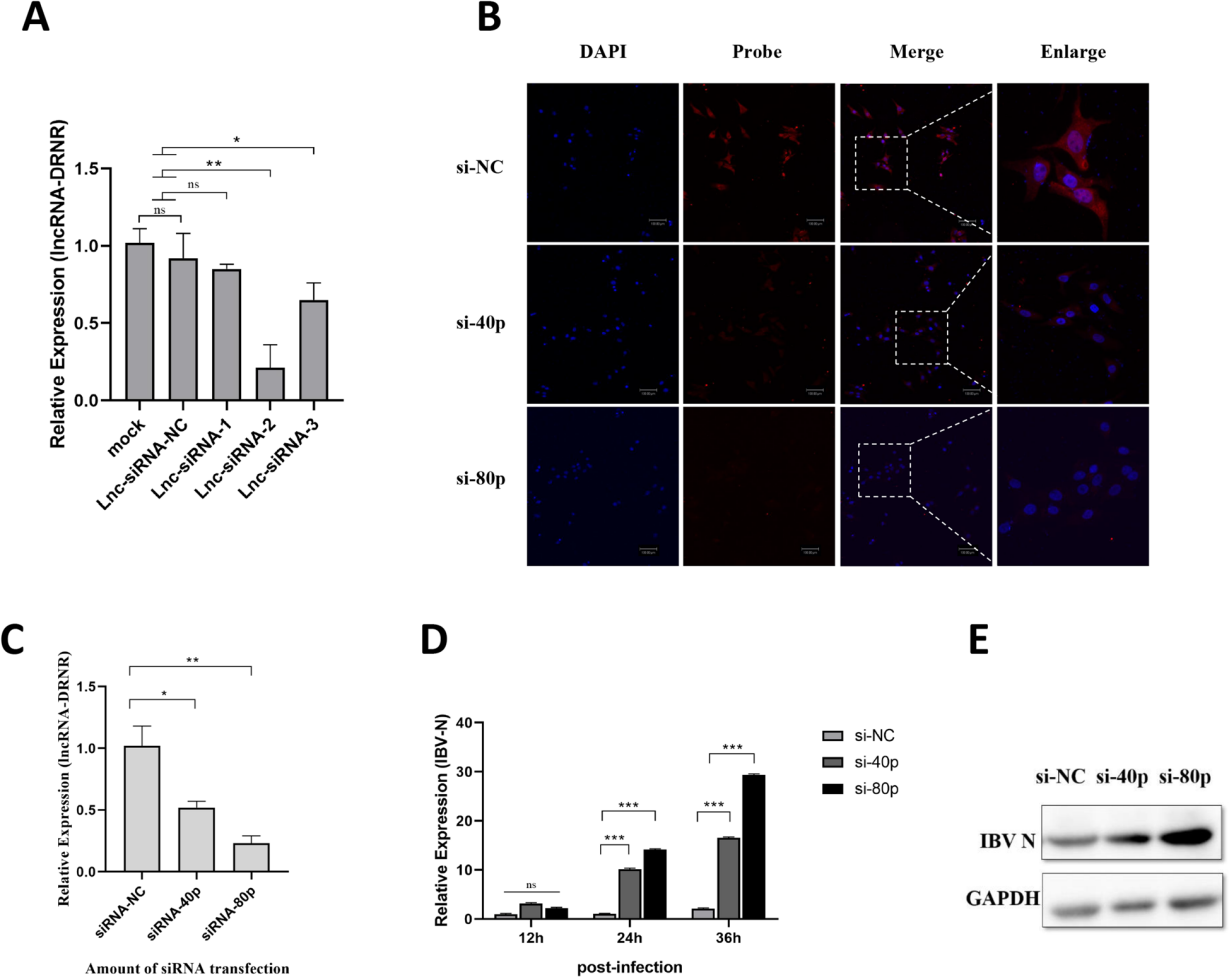
**Investigating the effect of siRNA-mediated knockdown of lncRNA-DRNR on IBV replication.** **A** Identification of the specific siRNA targeting sites for silencing lncRNA-DRNR. **B** As the amount of the siRNA transfection reagent increases, the fluorescence signal of lncRNA-DRNR weakens. **C** As the amount of the siRNA transfection reagent increases, the expression level of lncRNA-DRNR decreases. **D** Time-course and dose-dependent analysis of IBV-N mRNA levels following knockdown of lncRNA-DRNR using siRNA. **E** Impact of varying siRNA concentrations on the protein expression of the IBV-N viral protein.

To study the function of lncRNA-DRNR during IBV infection, we developed three siRNA sequences that specifically target the lncRNA-DRNR transcript. We transfected the HD11 cells with these three siRNAs and a negative control. We then assessed the knockdown efficiency using RT-qPCR at 36 hpi. The results indicated that lnc-siRNA-2 demonstrated the highest silencing efficiency, achieving approximately 76% knockdown. In contrast, lnc-siRNA-1 showed a 28% reduction, and lnc-siRNA-3 exhibited no inhibitory effect (Figure 3A). We used IFA to show that as the amount of si-lncRNA-DRNR transfection reagent increased, the fluorescence signal of lncRNA-DRNR decreased (Figure 3B), along with a gradual reduction in the expression level of lncRNA-DRNR (Figure 3C). Therefore, we selected lnc-siRNA-2 for further analysis. To examine the influence of lncRNA-DRNR on various stages of IBV replication, we transfected HD11 cells with lnc-siRNA-2 for 24 h, followed by IBV infection at an MOI of 2. We gathered cell samples at 12 hpi, 24 hpi, and 36 hpi, extracted total RNA, and measured the expression of the IBV N gene using RT-qPCR. The results showed that when lncRNA-DRNR was silenced, the expression of the IBV N gene increased significantly, suggesting that the down-regulation of lncRNA-DRNR effectively boosted IBV replication in HD11 cells (Figure 3D). Importantly, this boost was linked to the level of lncRNA-DRNR expression, as the enhancement was more noticeable when the amount of lnc-siRNA-2 used for transfection was increased from 40 to 80 pmol. Furthermore, we collected protein samples at the 36 hpi time point and performed WB analysis to identify the expression of the IBV N protein. The results indicated a significant increase in the expression level of the IBV N protein following the down-regulation of lncRNA-DRNR (Figure 3E).

### lncRNA-DRNR negatively regulates the expression of chJMJD6

**Figure 4:**
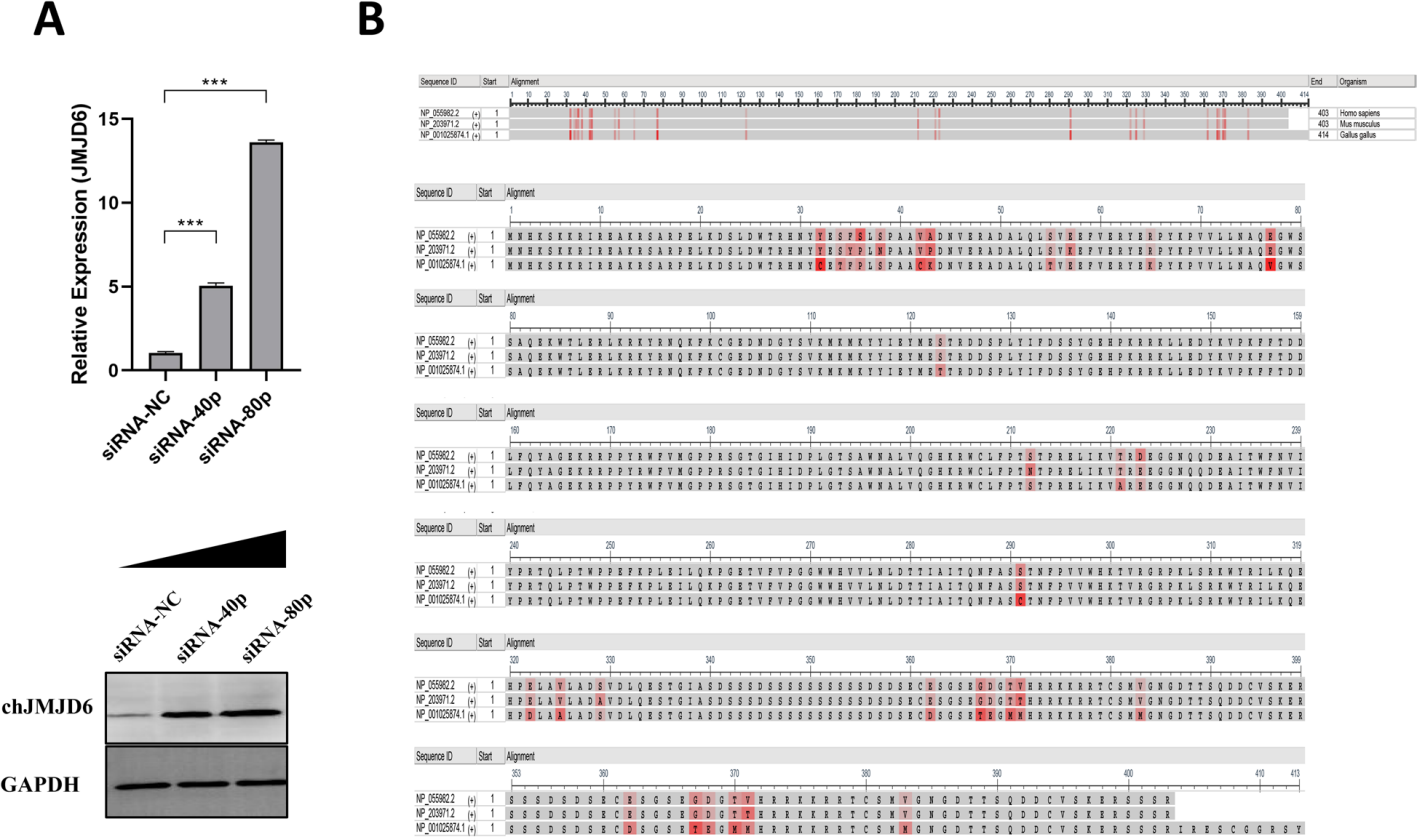
**Regulation of the target gene chJMJD6 by lncRNA-DRNR.** **A** Changes in the mRNA expression of the chJMJD6 following interference with different doses of siRNA-lncRNA-DRNR (up). Changes in the protein expression of the chJMJD6 following interference with different doses of siRNA-lncRNA-DRNR (down). **B** Comparative analysis of the chJMJD6 amino acid sequences across different species.

lncRNAs primarily regulate gene expression through cis or trans mechanisms rather than being translated into functional proteins like mRNAs. In our previous study, we identified potential target genes of lncRNA-DRNR by searching for protein-coding genes within a 100 kb genomic region upstream and downstream of the lncRNA locus. Among the candidate targets, chJMJD6 showed a relatively high Pearson’s correlation with lncRNA-DRNR, indicating it as a potential cis-regulatory target of this lncRNA. To further explore the regulatory relationship between lncRNA-DRNR and chJMJD6, we investigated the expression of chJMJD6 upon the down-regulation of lncRNA-DRNR. The results indicated that when the expression of lncRNA-DRNR decreased, the mRNA (Figure 4A up) and protein (Figure 4A down) expression of chJMJD6 showed a significant increase. This suggests that lncRNA-DRNR may have a negative regulatory effect on the expression of chJMJD6. Additionally, we compared the protein sequences of JMJD6 in various species, including humans (*Homo sapiens*, NP_055982.2), mice (*Mus musculus*, NP_203971.2), and chickens (*Gallus gallus*, NP_001025874.1), to investigate the potential of JMJD6 as a broad-spectrum antiviral therapeutic target. The analysis revealed that the amino acid sequence of chicken JMJD6 is more than 80% similar to the human and mouse counterparts, indicating a high level of conservation of this protein among these three species (Figure 4B).

### IBV can use chJMJD6 to promote its infection in HD11 cells

**Figure 5:**
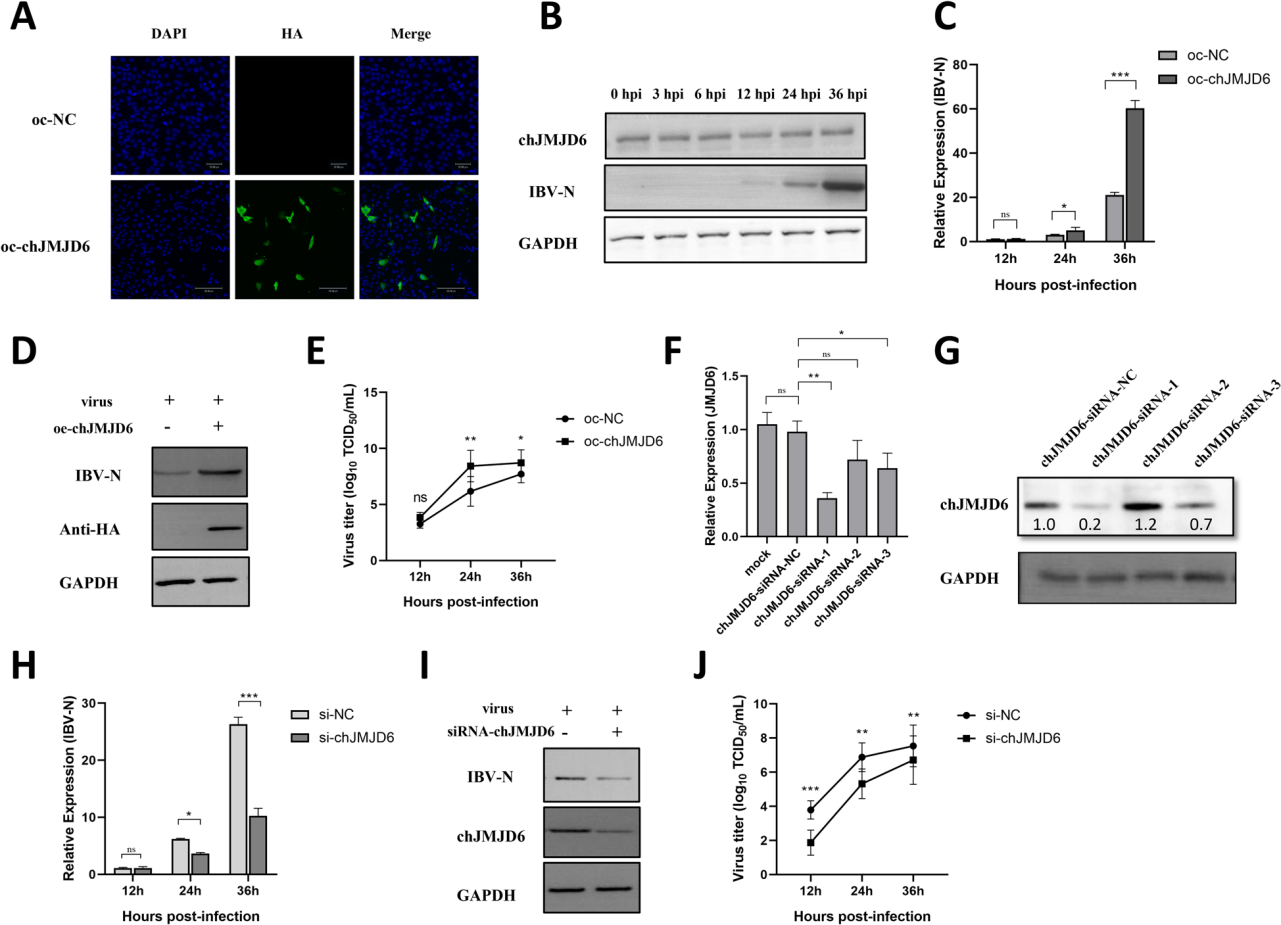
**Investigating the regulatory function of chJMJD6 in IBV infection.** **A** Quantitative analysis of chJMJD6 overexpression vector efficiency using indirect immunofluorescence. **B** Changes of chJMJD6 protein expression at different time points of IBV infection. **C** Time-course analysis of IBV replication upon chJMJD6 overexpression. **D** Protein expression analysis of IBV N protein following 36 h post-chJMJD6 overexpression. **E** The change in TCID_50_ of HD11 supernatant after infection with IBV following overexpression of chJMJD6; **F**–**G** Identification of critical chJMJ6 interference points through targeted siRNA interference screens; **H** Monitoring the effect of siRNA-mediated reduction of chJMJD6 expression on IBV-N mRNA levels at different time points post-infection. **I** Protein level assessment of IBV N by 36 hpi in siRNA-chJMJD6 knockdown samples. **J** The change in TCID_50_ of HD11 supernatant after infection with IBV following knockdown of chJMJD6 with siRNA.

To better understand the regulatory role of chJMJD6 in IBV infection, we created a eukaryotic expression vector for chJMJD6 and transfected it into HD11 cells. The outcomes revealed that cells containing the JMJD6 expression vector displayed intense fluorescence signals, suggesting successful overexpression of chJMJD6 (Figure 5A). Additionally, we collected infected cells at different time points (0 hpi, 3 hpi, 6 hpi, 12 hpi, 24 hpi, and 36 hpi) for WB detection. We found that the expression of JMJD6 protein did not change upon IBV infection (Figure 5B). We then investigated the impact of chJMJD6 overexpression on IBV replication. After overexpressing chJMJD6 and infecting the cells with IBV, we observed that the overexpression of chJMJD6 significantly enhanced IBV replication. This enhancing effect became more prominent as the infection progressed (Figure 5C). In line with this, western blot analysis showed a significant increase in the protein expression levels of IBV at 36 hpi (Figure 5D). Additionally, we developed three siRNAs (JMJD6-si-1, JMJD6-si-2, and JMJD6-si-3) targeting chJMJD6 based on its gene sequence (Gene ID: 417355). Transfection experiments demonstrated that chJMJD6-si-1 showed the most effective knockdown of JMJD6 expression at both the mRNA and protein levels (Figures 5E and F). When we used the most effective siRNA (chJMJD6-si-1) to reduce the expression of chJMJD6 and then infected the cells with IBV, we observed virus replication was impaired. This reduced replication state persisted until 36 hpi (Figure 5G). The observed changes in viral protein expression levels aligned with replication dynamics depicted in Figure 5H.

### chJMJD6 regulates the expression of IFN-β and interferon-stimulated genes

**Figure 6:**
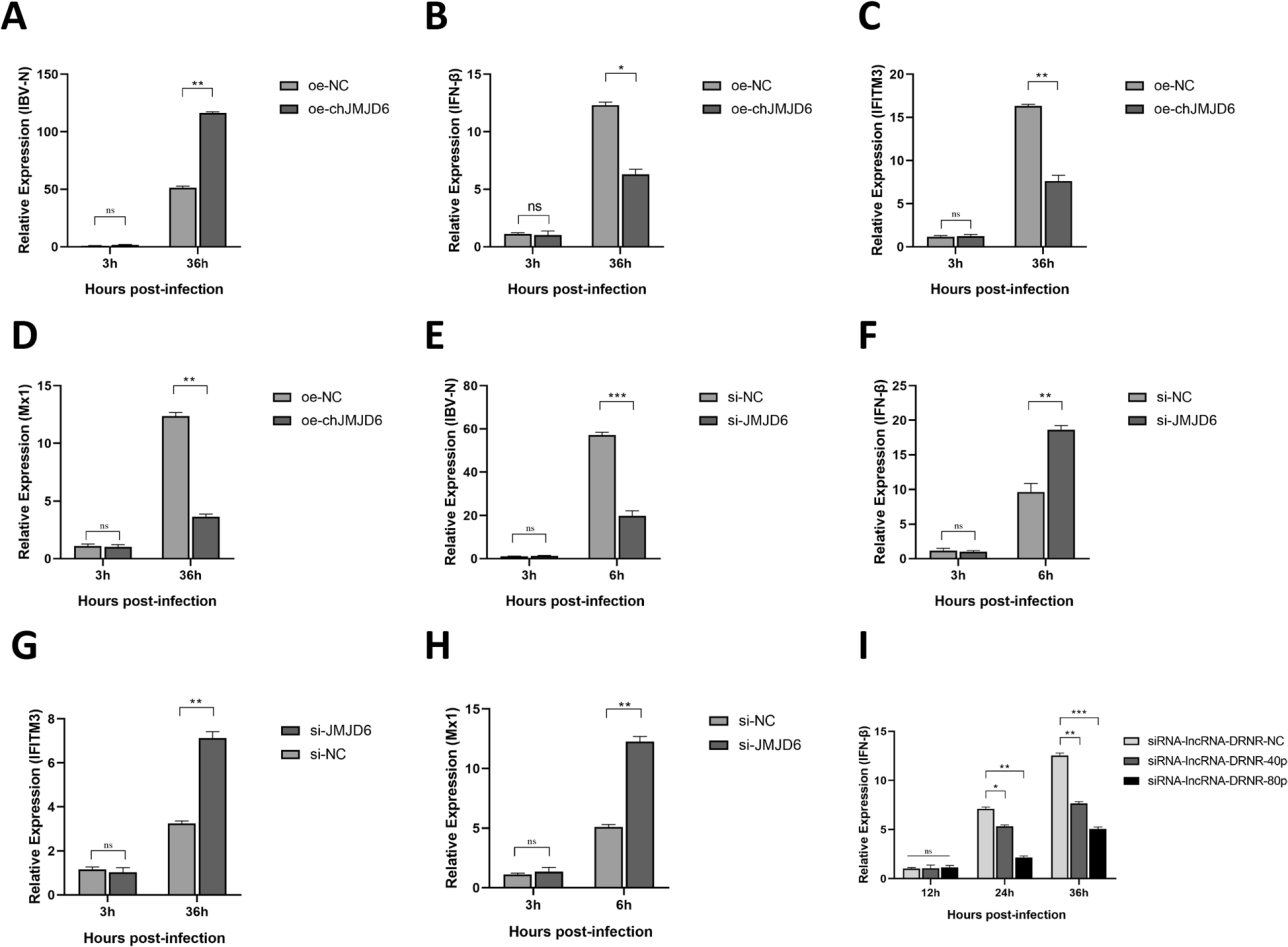
**Investigating the regulatory role of chJMJD6 in innate immune responses to IBV infection.** **A** Impact of chJMJD6 overexpression on the replication of IBV. **B** Upon overexpression of chJMJD6, the expression changes in IFN-β following IBV infection was analysed. **C** Upon overexpression of chJMJD6, the expression changes in IFITM3 following IBV infection were assessed. **D** Upon overexpression of chJMJD6, evaluation of the expression changes in Mx1 following IBV infection. **E** Effect of chJMJD6 knockdown using siRNA on the replication of IBV. **F** After silencing chJMJD6 expression, evaluation of the expression changes in IFN-βin response to IBV infection. **G** After silencing chJMJD6 expression, evaluation of the expression changes in IFITM3 in response to IBV infection. **H** After silencing chJMJD6 expression, evaluation of the expression changes in Mx1 in response to IBV infection. **I** Dose-dependent effects of siRNA-mediated knockdown of lncRNA-DRNR on the expression of IFN-β.

JMJD6 is a Jumonji C domain-containing protein family member and can function as an epigenetic regulator, demethylating its substrate proteins. JMJD6 can also demethylate TRAF6 to modulate toll-like receptor signalling [32]. Previous studies have shown that JMJD6 can reduce the production of IFN-I in response to Sendai virus (SeV) and vesicular stomatitis virus (VSV) infection. At the same time, JMJD6 inactivation enhances IFN-I production and impairs virus replication [33]. Given the critical role of JMJD6 in innate immunity, we sought to investigate whether the chicken homolog has a regulatory effect on the IFN-β signalling pathway triggered by IBV infection. Using RT-qPCR analysis, we found that overexpression of chJMJD6 at 36 h post-IBV infection significantly down-regulated the expression of the IFN-β gene (Figure 6B), as well as the expression of the interferon-stimulated genes IFITM3 (Figure 6C) and Mx1 (Figure 6D). In contrast, after chJMJD6 knockdown, the IBV-induced expression of the IFN-β gene at 36 hpi was significantly up-regulated (Figure 6F), and the expression of IFITM3 (Figure 6G) and Mx1 (Figure 6H) was also significantly up-regulated. Moreover, we observed decreased IFN-β expression in HD11 cells with increasing concentrations of siRNA-lncRNA-DRNR treatment (Figure 6I). These results suggest that the target gene chJMJD6 can suppress the expression of IFN-β, thereby inhibiting the downstream ISGs and promoting IBV replication. Importantly, the expression of the chJMJD6 gene is regulated by the lncRNA-DRNR.

### chJMJD6 Regulates STAT1 Methylation

**Figure 7:**
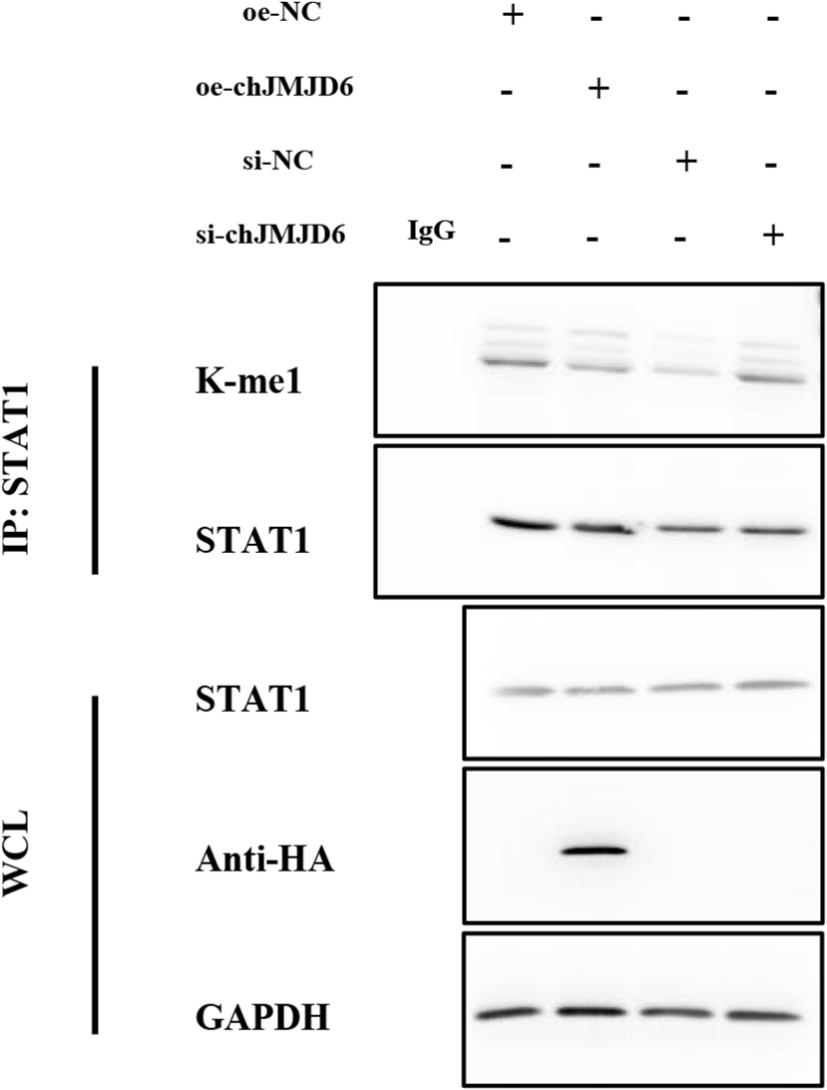
**Immunoprecipitation assay detecting the influence of altered chJMJD6 expression on STAT1 methylation.** Following overexpression of chJMJD6-HA (oe-chJMJD6) and siRNA knockdown of chJMJD6 (si-chJMJD6) in HD11 cells, a specific antibody for STAT1 was utilised for immunoprecipitation, with IgG serving as a control. WB detection was performed using antibodies targeting STAT1 and K-me1 methylation.

The methylation of STAT1 is essential for viral replication. JMJD6 can demethylate STAT1, reducing the signal transduction of the JAK-STAT pathway. This suppression further leads to a decrease in the activation of antiviral genes such as IFN-β and the expression of downstream interferon-stimulated genes [34]. To investigate the regulatory effect of chJMJD6 on STAT1 methylation, we performed co-immunoprecipitation and WB experiments to detect changes in STAT1 methylation status upon chJMJD6 overexpression or knockdown in HD11 cells. As shown in Figure 7, the overexpression of chJMJD6 in HD11 cells led to a decrease in the degree of STAT1 methylation. In contrast, the knockdown of chJMJD6 expression promoted STAT1 methylation, increasing the methylation level. These results demonstrate that chJMJD6 can regulate the methylation status of STAT1, which facilitates viral replication in the host cells. The ability of chJMJD6 to modulate STAT1 methylation provides a mechanistic explanation for its role in suppressing the IFN-β signalling pathway and promoting viral proliferation. These findings contribute to understanding the complex interplay between viral evasion strategies and the host's antiviral immune responses.

## Discussion

IBV was the first coronavirus discovered in nature, but effective means of inhibiting its replication have not been developed over the past decades [35]. The IBV genome is constantly mutating and reassorting, leading to the production of multiple serotypes. This results in poor cross-protection from vaccination [36]. Therefore, studying IBV infection pathogenesis and immunological mechanisms remains a crucial focus of IB prevention and control research. Extensive research has been conducted to investigate the host-virus interactions. LncRNAs have been found to participate in various physiological and pathological processes, making them promising therapeutic targets [37]. A groundbreaking study showed that lncRNA446 directly binds to the core component ALIX of the tight junction complex. It regulates the integrity of tight junctions and the degradation of ALIX by inhibiting its ubiquitination. This plays an essential role in resisting PEDV infection and providing new insights into the repair mechanism of coronavirus-induced intestinal barrier damage [38]. The previous studies observed that IBV infection can lead to changes in the lncRNA profile of the HD11. Notably, about 67% (122/181) of the detected lncRNAs showed a downward trend, suggesting that the stable expression of most lncRNAs is inhibitory to virus replication [29]. Among the down-regulated lncRNAs, we selected a significantly changed one, named lncRNA-DRNR, for further investigation. Interestingly, this lncRNA was not only considerably down-regulated in IBV-infected HD11 cells but also exhibited the same trend in two other cell lines, DF-1 and LMH, indicating that lncRNA-DRNR is likely a widespread IBV-regulatory lncRNA.

The regulatory potential of lncRNAs is closely related to their specific subcellular localisation within the cell, including the cell nucleus, chromatin, and cytoplasmic solvent. Some nuclear lncRNAs can influence chromatin structure and control transcriptional activity by interacting with chromatin regulatory proteins and promoting their recruitment or binding to chromatin [39, 40]. For instance, lnc-TCF7 promotes the transcription of the TCF7 gene by recruiting the SWI/SNF chromatin remodelling complex to the TCF7 promoter [41]. Similarly, the lnc-CAST recruits the histone acetyltransferase H3K27ac to activate the CXCL8 promoter, thereby enhancing the transcription of the CXCL8 gene [42]. Through FISH experiments, we have observed that the lncRNA-DRNR is localised in both the cell nucleus and the cytoplasm. The red fluorescence signal from the lncRNA-DRNR probe shows a good overlap with the DAPI-stained nuclear region. Furthermore, gene expression analysis by RT-qPCR on nuclear and cytoplasmic fractions reveals a relatively even distribution of lncRNA-DRNR between the two compartments. These findings suggest that lncRNA-DRNR is present in both the cytoplasm and the nucleus, indicating that it may play a role in chromatin's epigenetic modification and gene expression regulation. The modular and multi-chromosomal structural architecture of lncRNAs allows them to form conformational switches and interact simultaneously with mRNAs, DNA, and proteins, further expanding their regulatory potential [13, 43, 44].

JMJD6 belongs to the histone demethylase family and can catalyse the demethylation of arginine 2 on histone H3 and arginine 3 on histone H4 [45]. Previous studies have highlighted the role of JMJD6 in cancer, and its interactions with viruses are also gradually being uncovered. Zheng and others were the first to discover that JMJD6 helps to regulate the host's innate immune response, suppressing the production of IFN-I in host cells. They also elucidated that JMJD6 inhibits the IFN-I-induced antiviral response by promoting the degradation of activated interferon regulatory factor 3 (IRF3), thereby facilitating RNA virus replication [33]. Although the host utilises multiple mechanisms to resist IBV invasion, the overall clearance and suppression of IBV infection largely depends on the early protection provided by the innate immune system [46]. To survive and replicate successfully, IBV has evolved mechanisms to disrupt the activation of host antiviral signalling pathways, such as delaying the activation of the IFN response in the early stage of IBV infection [8]. Our data showed that overexpression of chJMJD6 suppressed the expression of the IFN-β gene, while knockdown of chJMJD6 promoted the expression of IFN-β. Additionally, some interferon-regulated genes, including IFITM3 and Mx1, were significantly down-regulated upon chJMJD6 overexpression but significantly up-regulated upon chJMJD6 knockdown. These results demonstrate that the chJMJD6 gene can suppress interferon expression, thereby inhibiting the activation of antiviral genes and ultimately promoting IBV replication. Tikhanovich et al. found that TRAF6, an essential mediator of the TLR pathway in the innate immune response, can be demethylated by JMJD6. The demethylation of TRAF6 by JMJD6 leads to its activation and up-regulation of the downstream NF-κB pathway [32]. Subsequently, Ganesan found that JMJD6-induced demethylation of STAT1 weakened the JAK-STAT signalling, thereby suppressing the activation of antiviral genes and ultimately enhancing HCV replication [34]. We then verified whether JMJD6 can regulate IBV replication similarly to HCV. We found that consistent with Murali’s study, overexpression of chJMJD6 inhibited the methylation of STAT1, while chJMJD6 knockdown had the opposite effect, promoting STAT1 methylation. However, the role of STAT1 methylation in IBV infection requires further investigation.

Our study focused on a long non-coding RNA, lncRNA-DRNR, which was found to be significantly down-regulated during IBV infection. We discovered that this lncRNA can negatively regulate virus replication. Further investigation revealed that the target gene of lncRNA-DRNR is chJMJD6, which was shown to inhibit the methylation of STAT1. This affected the induction of ISGs by IFN-β through the JAK-STAT signaling pathway, ultimately exerting an inhibitory effect on IBV replication. By targeting chJMJD6 and modulating STAT1 methylation, lncRNA-DRNR seems crucial in regulating the IFN-mediated antiviral signalling cascade. This regulation is essential for limiting IBV replication in the host. Further investigation into the molecular mechanisms involved will provide valuable insights into the host-virus interactions and potential therapeutic interventions against IBV infection.

## Data Availability

All data generated or analysed during this study are included in this published article and its supplementary information files.
